# Strategies for increasing participation in mail-out colorectal cancer screening programs: a systematic review and meta-analysis

**DOI:** 10.1186/s13643-019-1170-x

**Published:** 2019-11-04

**Authors:** Belinda C. Goodwin, Michael J. Ireland, Sonja March, Larry Myers, Fiona Crawford-Williams, Suzanne K. Chambers, Joanne F. Aitken, Jeff Dunn

**Affiliations:** 1 Institute for Resilient Regions, University of Southern Queensland, Springfield Central, QLD 4300 Australia; 2 School of Psychology and Counselling, University of Southern Queensland, Springfield Central, QLD Australia; 3 Cancer Research Centre, Cancer Council Queensland, Fortitude Valley, QLD Australia; 4 Faculty of Health, University of Technology Sydney, Sydney, Australia; 5 Prostate Cancer Foundation of Australia, St Leonards, NSW Australia; 6 Menzies Health Institute, Griffith University, Southport, QLD Australia; 7 School of Public Health and Social Work, Queensland University of Technology, Brisbane, QLD Australia; 8 Menzies Health Institute Queensland, Griffith University, Brisbane, QLD Australia; 9 School of Social Science, University of Queensland, Brisbane, QLD Australia; 10 School of Medicine, Griffith University, Brisbane, QLD Australia

**Keywords:** Fecal occult blood test, Colorectal cancer screening, Bowel cancer screening, Population screening, Intervention, Systematic review, meta-analysis

## Abstract

**Background:**

Population mail-out bowel screening programs are a convenient, cost-effective and sensitive method of detecting colorectal cancer (CRC). Despite the increased survival rates associated with early detection of CRC, in many countries, 50% or more of eligible individuals do not participate in such programs. The current study systematically reviews interventions applied to increase fecal occult blood test (FOBT) kit return, specifically in population mail-out programs.

**Methods:**

Five electronic databases (PubMed, PsycINFO, Scopus, CINAHL, and ProQuest Dissertations and Theses) were searched for articles published before the 10th of March 2018. Studies were included if they reported the results of an intervention designed to increase the return rate of FOBT kits that had been mailed to individuals’ homes. PRISMA systematic review reporting methods were applied and each study was assessed using Cochrane’s Risk of Bias tool. Pooled effect sizes were calculated for each intervention type and the risk of bias was tested as a moderator for sensitivity analysis.

**Results:**

The review identified 53 interventions from 30 published studies from which nine distinct intervention strategy types emerged. Sensitivity analysis showed that the risk of bias marginally moderated the overall effect size. Pooled risk ratios and confidence intervals for each intervention type revealed that telephone contact RR = 1.23, 95% CI (1.08–1.40), GP endorsement RR = 1.19, 95% CI (1.10–1.29), simplified test procedures RR = 1.17, 95% CI (1.09–1.25), and advance notifications RR = 1.09, 95% CI (1.07–1.11) were effective intervention strategies with small to moderate effect sizes. Studies with a high risk of bias were removed and pooled effects remained relatively unchanged.

**Conclusions:**

Interventions that combine program-level changes incorporating the issue of advance notification and alternative screening tools with the involvement of primary health professionals through endorsement letters and telephone contact should lead to increases in kit return in mail-out CRC screening programs.

**Systematic review registration:**

This review is registered with PROSPERO; registration number CRD42017064652

## Background

Colorectal cancer (CRC) is a leading cause of morbidity and mortality for men and women internationally, accounting for 9% of all cancer incidence and 8% of all cancer deaths [1]. For this reason, several nations throughout the world have implemented large-scale population CRC screening campaigns in order to increase early detection and thereby improve survival. One increasingly utilized method of conducting wide-spread CRC screening is to mail self-administered fecal occult blood test kits (hereon FOBT) to all older (and therefore at higher risk) adults (e.g., 50–74 year olds) with instructions for stool sample collection and return. FOBT mail-outs are a convenient, cost-effective, and sensitive method of increasing early detection of CRC [2, 3] and participation in such programs is associated with earlier detection and reduced CRC mortality [4–7].

Unfortunately, despite the substantial increase in CRC survival rates associated with early CRC detection, individual participation (i.e., valid and complete kit return) remains below 50% in the majority of participating nations including Australia, France, Czech Republic, Germany, Latvia, and Croatia [8, 9]. For this reason, health professionals and researchers are experimenting with strategies to increase participation in mail-out FOBT screening campaigns. A systematic review of all the interventions that have been implemented to increase FOBT kit return, specifically in population mail-out screening programs, has not been reported. This study aims to determine which interventions are most supported in the literature and which have the largest effects. To do this, the current study systematically reviews all published studies where interventions have been applied to mail-out FOBT kits and kit return has been reported as an outcome.

## Method

### Search strategy

**Fig. 1:**
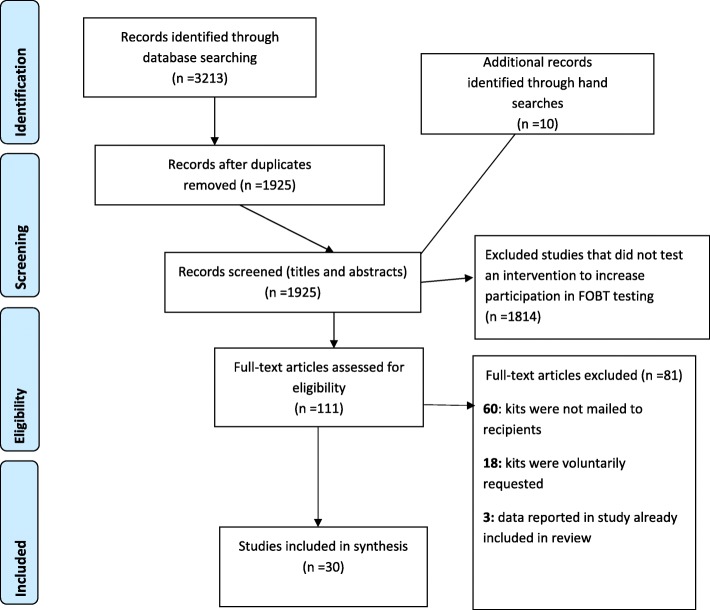
PRISMA flow chart of search and filter results

A systematic review was conducted to identify studies (published before the 10th of March 2018) in which an intervention to increase participation in a mail-out FOBT-based CRC screening program was assessed. The review methodology followed the PRISMA statement for the conduct and reporting of systematic reviews [10] (Fig. 1 contains PRISMA flow chart and PRISMA checklist is available as Additional file 1). The review protocol was registered with International Prospective Register of Systematic Reviews (PROSPERO); registration number CRD42017064652. Five electronic databases, PubMed, PsycINFO, Scopus, CINAHL, and ProQuest Dissertations and Theses A & I, and Google Scholar were searched using title, abstract, and keyword searches for terms such as “fecal (and faecal) occult blood,” “FOBT,” “uptake,” “participation,” and “compliance” (see Additional file 2 for precise syntax). The initial database search was conducted on 6 March 2017 by the first author. Hand searches for relevant articles were also conducted, through ancestral searching of the reference lists of included articles, as well as searching included articles in Google Scholar and using the “cited by” function. This exact search strategy was replicated by a second independent reviewer between the 17th and 31st of May 2017 with no new articles identified. The search was replicated a third time by a third reviewer (LM) between the 10th and 24th of March 2018 to ensure our results were as up to date as possible before publication, and two further studies eligible for inclusion were identified in this final search [16.45]. The search results detailed in the systematic review findings and the PRISMA flow chart in Fig. 1 reflect this latest search.

### Study selection

To be included in the current review, articles needed to involve the implementation of a strategy to increase the return rate of FOBT kits in a postal CRC screening program. Only quantitative studies where the outcome variable was the return of FOBT kits were included. Articles were excluded if test kits were voluntarily requested, not routinely mailed to participants, where the return of FOBT kits was not reported separately from other methods of CRC screening, or if the data had been reported in another study already included in the review. Review articles, conference abstracts, and articles not available in the English language were also excluded. Full-text screening of potentially relevant articles was carried out using these criteria. As with the searches, an independent reviewer replicated this study selection protocol in May 2017 and a third reviewer (LM) conducted the study selection protocol again on the updated search results in March 2018. The results pertaining to the study selection detailed in the systematic review findings and the PRISMA flow chart in Fig. 1 reflect the latest search.

### Analysis

Summary data was extracted for each intervention, including author, sample size, age and country of participants, intervention type, comparison group, and key findings. These are presented in Additional file 3. Unadjusted risk ratios (RR) and 95% confidence intervals (CI) for each intervention were calculated using raw, intention to treat (where available), uptake data provided within each article. The Cochrane Risk of Bias tool [11] was applied to each study by the first author, with outcomes replicated by an independent reviewer without discrepancy. Sources of bias such including, randomization, allocation, blinding, incomplete outcome data, and selective reporting were graded as low, high risk, or unclear for each study. Studies with at least one element assessed as high risk were rated as “high risk of bias” and studies with two or more elements assessed as unclear were rated as “unclear risk of bias,” while the remainder of studies were rated as “low risk of bias.” A total pooled effect size was calculated with the risk of bias as a moderator for a sensitivity analysis. Pooled effect sizes (RR) and 95% CIs are presented and compared for each intervention category with three or more studies. Data analysis was conducted in R using the Metafor package [12, 13]. Heterogeneity was assessed by calculating *I*^2^ values. Due to variations in populations and implementation of interventions within each category, random effects models with restricted maximum likelihood estimation were utilized [14, 15]. Publication bias (and other small study effects) was assessed quantitatively via inspection of funnel plots and consulting Egger’s regression test for funnel plot asymmetry.

## Results

### Systematic review

As detailed in Fig. 1, 3213 records were identified in the initial search. After duplicates were removed, records were screened for relevance first by reviewing titles and abstracts, leaving 111 articles. This included an additional 10 records that were identified through hand searches during this process. From this, 81 articles were excluded based on the inclusion-exclusion criteria described above, leaving 30 for inclusion in the current systematic review. Study origins, dates, designs, effect sizes, and conclusions from 59 interventions within the 30 studies reviewed are detailed in the data extraction table Additional file 3. These studies originated from 10 different countries including Australia (*n* = 7), UK (Scotland, North Ireland, Wales, and England; *n* = 13), USA (*n* = 3), Netherlands (*n* = 4), Israel (*n* = 1), Spain (*n* = 1), and Latvia (*n* = 1). Sample sizes for each intervention ranged from 153 to 1,167,017 (median = 5000). As detailed in Table 1, nine distinct intervention types were identified based on the strategies they employed.

**Table 1:** Types of interventions

Category	*n*	Description
Behavior priming	6	Where the text content within printed materials accompanying a kit were manipulated to prime or encourage screening behavior (e.g., priming regret, implying advocacy from others, setting intentions).
Added print materials	11	Where extra information in print format was added to the standard kit (e.g., educational booklets on CRC, or enhanced instructions).
Incentive	1	Where participants were offered an incentive to return completed kits.
Advance notification	4	Where letters notifying participants of the kit arrival were sent out prior to the kit.
Outdoor advertising	1	The placement of billboards or posters in public areas.
Simplified test	17	Where the FOB testing procedure itself was simplified or enhanced for the user (e.g., removing dietary restrictions or including collection papers).
Telephone contact	4	Where participants were contacted by telephone for the reminder and/or instruction purposes.
GP endorsement	6	Where the kit invitation included a letter from the participant’s GP or GP practice.
Digital reminder	3	Where participants are sent either an SMS or email reminder to complete and return FOBT kit

### Risk of bias

As detailed in Table 2, studies were largely rated as having a low risk of bias (*n* = 29). Ten studies had an unclear risk of bias and four studies had a high risk of bias. Sixteen of the studies reviewed did not provide details within the manuscript regarding whether efforts were made to blind research personnel or participants to group allocation, and 1 study indicated that blinding of personal and participants did not occur. Ten studies neglected to report whether the group allocation process was concealed from research personnel and two studies indicated this did not occur. In 12 studies, it was unclear whether selective reporting had occurred as a clear outcome variable was not defined (e.g., whether participation was measured on an “intention to treat basis”). Eight studies did not describe the procedure for random allocation, while two studies indicated that participants were not randomly allocated to the intervention group. In terms of the separate interventions, 11 interventions came from high risk of bias studies, 22 from unclear risk studies, and 26 from low-risk studies. Risk of bias within each intervention type is reported within the systematic review findings and tabled in Additional file 3.

**Table 2:** Cochrane Collaboration tool for assessing risk of bias

First author and year	Random sequence generation	Allocation concealment	Blinding of participants and personal	Blinding of outcome assessment	Incomplete outcome data	Selective reporting	Other bias	Overall Risk of Bias
Benton et al. [16]	H	H	H	L	L	L	L	H
Cole et al. [17]	L	L	U	L	L	L	L	L
Cole et al. [18]	L	L	L	L	L	L	L	L
Cole et al. [19]	U	U	U	L	U	L	U	U
Corondado et al. [20]	L	U	U	L	L	L	L	L^#^
Denters et al. [21]	L	L	U	U	L	U	U	U
Deutekom et al. [22]	L	L	L	U	L	L	L	L
Gupta et al. [23]	L	L	L	L	L	L	L	L
Hewitson et al. [24]	L	L	L	L	L	L	L	L
Hirst et al. [25]	L	L	L	L	L	U	L	L
Hughes et al. [26]	H	H	U	U	H	U	H	H
King et al. [27]	U	U	U	L	L	U	H	H
King et al. [28]	U	U	U	L	L	U	L	U
Libby et al. [29]	L	L	L	L	U	L	L	L
Lo et al. [30]	L	U	U	L	L	U	L	U
McGregor et al. [31]	U	L	L	L	L	L	L	L
Moss et al. [32]	L	L	U	L	L	L	L	L
Myers et al. [33]	U	U	L	L	U	L	L	U
Neter et al. [34]	L	L	U	L	L	L	L	L
O'Carroll et al. [35]	L	L	U	L	L	L	L	L
Robinson et al. [36]	U	U	U	L	L	U	L	U
Santare et al. [37]	L	L	U	U	L	U	L	U
van Roon, [38]	L	L	U	L	U	U	L	U
van Rossum et al. [39]	L	L	L	U	L	L	L	L
Verne et al. [40]	U	U	L	U	L	U	U	U^^^
Wardle et al. [41] (1)	L	L	L	L	L	L	L	L
Wardle et al. [41] (2)	L	L	L	L	L	L	L	L
Wardle et al. [41] (3)	L	L	L	L	L	L	L	L
Wardle et al. [41] (4)	L	L	L	L	L	U	U	L
Watson et al. [42]	L	U	U	L	L	L	L	L
White et al. [43]	U	U	U	L	L	L	L	U^*^
Zajac et al. [44]	L	L	L	U	L	L	H	H^¥^
Zubero et al. [45]	L	L	L	L	L	U	L	L

*L* = low, *U* = unclear, *H* = high

^#^Risk of bias is high for the email intervention as not randomly assigned

^Risk of bias is suggested to be high for the self- versus lab-analyzed stool sample intervention as it is much less likely that participants would return self-analyzed negative results

*Risk of bias is suggested to be high for the outdoor advertising intervention implemented in White et al. 2015 because participants not randomly allocated

^¥^Zajac et al., 2010 reports on the same sample as Cole et al., 2007 and was therefore deemed high risk of bias

### GP endorsement

Twelve interventions reported across six studies [16, 17, 27, 44, 46, 47] from Australia and the UK utilized GP endorsement. Eight of the interventions were from high risk of bias studies and four were from low-risk studies. Eight of these interventions were from two studies that trialed two interventions at four time points on the same sample (accounting for the large proportion of high risk of bias interventions in this group). Eight interventions tested the effect of an endorsement letter personally signed by the participant’s GP, all of which were significantly effective in increasing screening uptake with effect sizes ranging from RR = 1.01 (CI = 1.01–1.02) to RR = 2.19 (CI = 1.67–2.87). The remaining interventions tested the effectiveness of including a letter from the GP practice (rather than from the specific GP) over four time points, showing a statistically significant positive effect on uptake at the first RR = 1.27 (CI = 1.12–1.44) and fourth RR = 1.19 (CI = 1.04–1.37) time point.

### Behavior priming

Six interventions from five studies in Australia, UK, Israel, and the USA utilized behavior priming as an intervention strategy [18, 30, 33, 35, 48]. Two interventions were from low risk of bias studies and four were from the unclear risk of bias studies. Only one intervention, using an intention implementation technique, resulted in a significant increase in uptake of screening, RR = 1.05 (CI = 1.04–1.07). In this intervention, prewritten if-then statements regarding when, where, and how to take the test were included with the FOBT kit [48]. Interventions utilizing priming techniques such as advocacy from other people, information on risks, and the priming of anticipated regret did not yield significant effects.

### Print materials

Eleven interventions from nine studies across Australia, the UK, and the USA added printed content to the screening kit [29, 31, 33, 35, 42, 43, 46, 47, 49]. Three of the interventions came from studies with an unclear risk and the remainder was from low-risk studies. One intervention that issued a leaflet with enhanced “easy to read” instructions and a CRC educational component showed a small significant increase in kit return [46], RR = 1.12 (CI = 1.01–1.23). Another issuing an enhanced reminder letter [47] had a very weak significant positive effect of uptake, RR = 1.03 (CI = 1.01–1.05). Of the five interventions that involved sending CRC information booklets with kits, none showed a significant effect on uptake. Two interventions that included surveys into the invitation packs had no significant effect on uptake [35, 42] and the inclusion of narrative leaflets (*n* = 2) were associated with very small *decreases* in participation [31, 42].

### Simplified test procedures

Seventeen interventions from 12 different studies based in Australia, Netherlands, the UK, Latvia, and Spain involved simplifying test procedures [18, 21, 22, 26, 36, 37, 39, 40, 43, 45, 50]. Two of the interventions were from high-risk studies, nine were from studies with an unclear risk of bias, and six were from low-risk studies. The provision of an immunochemical (FIT) rather than a guaiac (gFOBT)-based test kit (usually involving fewer stool samples and/or the removal dietary restrictions) had a significant positive effect in all five trials, with effect sizes ranging from RR = 1.12 (CI = 1.11–1.13) to RR = 1.48 (CI = 1.41–1.55). Of the three interventions, that only removed dietary restrictions from kit instructions, two showed significant positive effects on uptake [18, 36] RR = 1.42 (CI = 1.09–1.83) and RR = 1.50 (CI = 1.27–1.76), whereas the other showed no significant effect [40]. Interventions that involved enclosing gloves with the stool collection kit [43] or providing a potentially superior brand of FIT kit [18, 37, 45] also yielded significant positive effects on kit return with effect sizes ranging from RR = 1.05 (CI = 1.01–1.10) to RR = 1.47 (CI = 1.28–1.68). Interventions that did not have significant positive effects on uptake included the issue of a kit where samples were self-analyzed at home and those where stools sample was collected using a or feces collection paper [21, 40].

### Telephone contact

Of the four interventions that applied telephone contact strategies (two each from two USA-based studies, one unclear and one low risk of bias) [33, 51], three reported significant positive effects on uptake with effect sizes ranging from RR = 1.29 (CI = 1.12–1.49) to RR = 1.34 (CI = 1.13–1.60). These three studies included two live 30-day reminder telephone call interventions [33, 52] and one intervention where kit recipients received an instruction call to assist with completing the kit [33]. The telephone intervention that had no effect on uptake involved an automatic reminder phone call [51].

### Advance notification

Four interventions from four studies based in Australia, the UK, Latvia, and the Netherlands, issued advance notification letters to participants approximately 2 weeks prior to sending the invitation and FOBT kit [19, 29, 37, 38]. Two interventions from studies with an unclear risk of bias and two were rated as low. All had a significant positive effect on the uptake, with effect sizes ranging from RR = 1.06 (CI = 1.01–1.11) to RR = 1.22 (CI = 1.08–1.39).

### Digital reminders

Three interventions utilizing digital reminders from two studies in the UK and the USA were identified where text messages or emails were sent to participants [51, 53]. None of these interventions were successful in significantly increasing participation. The two interventions utilizing text messages came from studies with a low risk of bias, whereas the email intervention was rated as a high risk of bias intervention due to non-random group allocation. Interventions using text messages did not show significant negative effects overall, but in a sub-sample of previous non-responders, a text message from a GP was associated with a significant increase in program uptake in one study [53] RR = 1.16 (CI = 1.02–1.32).

### Other interventions

One intervention from a low risk of bias USA study offering small ($5–$10) monetary incentives to participants [23] did not report a significant increase in screening uptake, and one high risk of bias campaign involving outdoor advertising, posters, and GP and adverts on pharmacy bags [43] had a significant positive effect on uptake RR = 1.08 (CI = 1.05–1.14).

### Meta-analysis

**Fig. 2:**
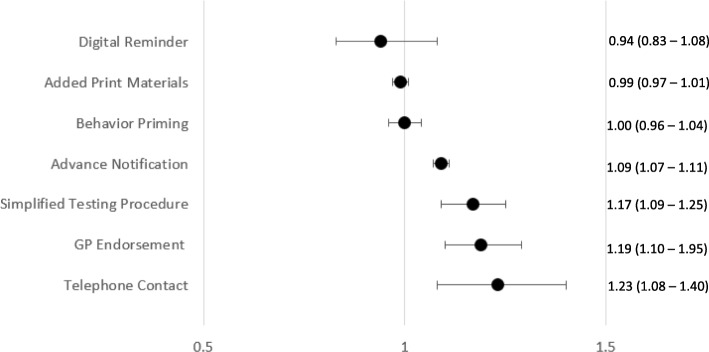
Pooled risk ratio estimates and 95% confidence intervals for each intervention type (interventions from high risk of bias studies included)

**Fig. 3:**
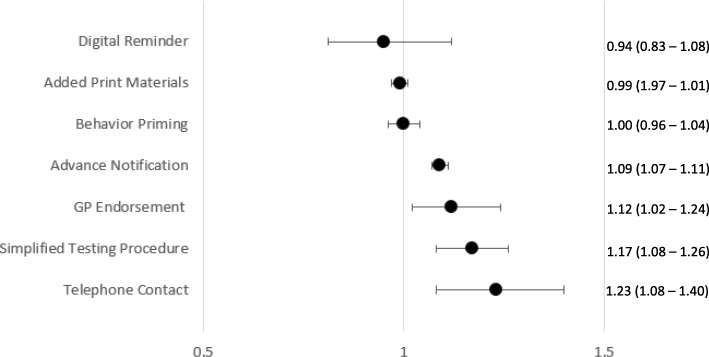
Pooled risk ratio estimates and 95% confidence intervals for each intervention type (interventions from high risk of bias studies not included)

Pooled effect sizes for each intervention type (excluding those with only one intervention) were calculated and presented in Figs. 2 and 3. When pooled effect sizes for each intervention type were compared, telephone contact RR = 1.23 (1.08–1.40) was associated with the highest increase in uptake, followed by GP endorsement RR = 1.19 (1.10–1.29), simplified test procedures RR = 1.17 (1.09–1.25), and advance notification RR = 1.09 (1.07–1.11). This translates to approximately 5 to 7% more kits being returned kits in intervention groups compared to reference groups. Forest plots for each intervention type are provided in Additional file 4.

### Sensitivity analysis

The moderation effect of risk of bias was marginal (*Q* (2) = 5.54, *p* = .06) with interventions from the high risk of bias studies yielding a slightly higher effect RR = 1.20 (CI = 1.11–1.30) than interventions low-risk studies RR = 1.08. (CI = 1.03–1.13). The pooled RR of interventions from unclear studies RR = 1.08 (1.02–1.14) did not differ significantly from interventions from high or low-risk studies. The moderation effect of type of intervention was significant *Q* (9) = 78.41, *p* = .001. Interventions from studies with a high risk of bias were removed and pooled effects for each intervention type were recalculated. These effect sizes were almost identical, with the key exception that the pooled estimate for GP endorsement decreases to RR = 1.12 (1.02–1.24). Separate plots for each intervention type can be found in Additional file 4.

Post hoc meta-analysis including only these four intervention types showed that intervention type was not a significant moderator of effect size *QM* (3) = 1.46, *p* = 0.70, suggesting they are statistically, equally effective. Pooled effects of behavior priming RR = 1.00 (0.96–1.04) digital reminders RR = 0.94 (0.83–1.08), and print materials RR = 0.99 (0.97–1.01) were not significantly different from zero.

### Heterogeneity

Without moderators in the model, heterogeneity was high, with 97.2% of dispersion attributable to between-study differences. Heterogeneity remained high within each risk of bias group, *I*^2^_high_ = 80.01%, *I*^2^_unclear_ = 98.54%, *I*^2^_low_ = 93.14%. Heterogeneity levels varied widely between intervention types, ranging from *I*^2^ = 98.5% and *I*^2^ = 95.6% for simplified test procedures and GP endorsement respectively to *I*^2^ = 1.1% for advance notifications. Added print materials and behavioral priming both had *I*^2^ values of 85.5% while telephone contact and digital reminders were *I*^2^ = 61.5% and *I*^2^ = 53.9% respectively.

### Publication bias

There was marginal evidence of publication bias *t* (57) = 2.07, df = 57, *p* = 0.04. The King et al. 1992 study trialing a GP endorsement was an outlier in terms of effect size (RR–2.19) and standard error (low). With this influence removed, publication bias decreased significantly *t* (56) = 1.94, *p* = 0.06. Publication bias within each intervention type was not evident (all *p*s < .05) except for GP endorsement *t* (10) = 6.07, *p* < .01. This was also likely due to the influence of the King et al. 1992 study. Pooled effect sizes from GP endorsement were presented with and without this intervention which also had a high risk of biased results according to the Cochrane Risk of Bias evaluation.

## Discussion

Several effective intervention strategies for increasing kit return in mail-out, population-based FOBT programs were identified through this systematic review and meta-analysis. The evidence reviewed here provided support for the use of four key intervention strategies including advance notification, GP endorsement, telephone contact, and the simplification of testing procedures. These findings have implications for both policy change and practical program changes that may increase future uptake in population mail-out CRC screening campaigns.

Although there were no statistically significant differences in effectiveness between the four key identified strategies, interventions involving telephone contact were associated with the highest average increase in the rate of kit. Limited studies (*n* = 2) have trialed the use of telephone contact as an intervention strategy; however, the available evidence suggests that live phone calls with kit recipients are associated with the higher kit return. Automated phone calls, where the recipient is played a recorded message, are not [52]. Interestingly, other forms of automated contact such as email and text message reminders were also unsuccessful, in one instance leading to decreased participation [52], suggesting that human interaction might be key to the effectiveness of personal reminders.

GP endorsement is a well-researched intervention strategy in terms of both the amount and consistency of evidence available, providing sound justification for its application in future CRC screening initiatives. Even when interventions from studies with high risk of bias were ignored, the inclusion of GP endorsement letter with the FOBT kit was associated with a modest, but significant, increase in kit return. These findings are not surprising considering GP information is the most frequently sought and trusted source of medical advice for most people [54–56] and the demonstrated positive effect of GP involvement in increasing cancer screening in general [57].

According to the current review, telephone contact and GP endorsement are both promising strategies for increasing FOBT kit return, however, the underlying reasons for their success remain unknown. For example, GP endorsement may be effective due to the participant having a personal (i.e., face-to-face connection) with their GP, the respect they have for their GP, or due to the letter being personally signed by someone. In the current review, interventions where letters were signed by the GP (as opposed to the GP practice more broadly) demonstrated higher and more consistent positive effects on kit return. In conjunction with the finding that live telephone calls were more effective than automated reminders, this suggests that a key element of intervention success may be a personal or proximal connection with the sender/caller. However, the effectiveness of these strategies may also be moderated by factors such as participant trust in the organization contacting them or the degree to which they generally adhere to advice from health professionals. Studies reviewed here that reported on the effects of GP endorsement were conducted in communities where general practice is a strong component of the health system. In settings where this is not the case, the effectiveness of GP endorsement would likely be less, and endorsement of CRC screening may be more effective coming from other respected sources of health advice. Future qualitative and experimental research can help draw conclusions about the mechanisms through which GP endorsement and telephone contact might be effective as well as the context in which these strategies will be most successful.

Simplification of the testing procedure also tended to be associated with the increased kit return. Hesitation or refusal to partake in CRC screening has been associated with reactions of disgust at the thought of stool manipulation [58]. Interestingly, however, interventions where extra or modified materials were supplied to assist with stool collection and limit contact (e.g., including stool collection wipes or collection papers) were generally not effective. Instead, interventions that reduced the number of steps or the effort required to take the test were more often associated with kit completion and return. For example, the removal of dietary restrictions and issue of immunochemical-based (FIT; 2 sample, with no diet restriction) rather than guaiac-based (gFOBT; 3 sample, with diet restrictions) kits tended to be most consistently effective in increasing kit return and with the largest effects.

Providing participants with advance notification before sending the kit—a practice already standard in many programs—had a consistent, but small effect across the four trials reviewed. Agencies not already issuing advance notifications to potential participants may see modest increases in participation by adopting this method.

Studies that assessed the use of print materials and behavior priming techniques were generally unsuccessful, suggesting the actual content of the printed materials accompanying kits appeared to have less impact on uptake than the source of the content (i.e., GP signed). Strategies that involved adding extra print materials to FOBT kits were largely unsuccessful in encouraging participation. In fact, the inclusion of a narrative information leaflet had a negative effect on kit return in two separate studies, suggesting that excessive information may burden and deter, rather than encourage, participation. The manipulation of text within print materials to prime screening behavior was also, for the most part, unsuccessful. However, only a small group of behavior priming strategies have been trialed. These include highlighting the negative consequences of inaction, intention implementation, and advocacy from others. There is still scope for research into different types of behavior priming interventions and definitive conclusions cannot yet be drawn about the effectiveness of this type of intervention strategy broadly. Nevertheless, according to the evidence reviewed, adding or manipulating printed content has not been supported as a successful intervention strategy.

Other intervention types were underrepresented in the available literature and consequently, the degree to which they are effective is unknown. Our understanding of the effectiveness of outdoor advertising may be strengthened in the future by implementing better controlled experimental designs. Intriguingly, offering a monetary incentive did not show promising results in the current review. No studies examined the use of social media or media campaigns.

### Policy and practical implications

Given that mail-out FOBT screening campaigns tend to be population-wide, government-driven initiatives, the current findings have several policy-level implications. For example, the FIT has been adopted by the majority of screening programs internationally; however, programs in some countries including Croatia, Finland, Portugal, and China still distribute the traditional gFOBT kits [9, 59]. Policy-makers in these nations may see a rise in CRC screening and detection rates should they adopt the FIT as their standard screening tool [8, 59]. Similarly, incorporating advance notification as part of the standard protocol for kit distribution should result in small increases in uptake. The current review suggests that resources spent on designing and distributing extra print materials with FOBT kits might be more effectively spent on other intervention methods.

Interventions that involve GPs or other professional endorsement of screening can be affected at a practical level. For example, health professionals and organizations may be able to contribute to increased uptake in CRC screening by contacting patients eligible to receive kits by phone, mail, or in person to encourage screening and provide support during the testing process.

Ultimately, an intervention that combines program-level changes and the involvement of primary health services may potentially have the most positive effect on uptake. For example, substantial increases in participation could result if kit simplification and endorsement strategies are combined, increasing both intentions to screen and the time and resources one has to complete the test. However, the additive benefits of such approaches have not yet been empirically tested.

### Strengths and limitations

This systematic review is the first to focus specifically on methods of increasing uptake in CRC screening via mail-out FOBTs, providing up to date and rigorously obtain information regarding the most effective interventions specific to this form of a public health initiative. However, some limitations apply. Although a comprehensive list of relevant databases was searched, the list was not exhaustive and more articles may have been identified if other databases such as Embase or CENTRAL were included. Heterogeneity across interventions was high even within categories; likely reflecting varying methodologies applied in each. It is also important to note that *I*^2^ values are susceptible to over-estimation in large sample sizes and therefore must be interpreted with caution [60]. Nevertheless in the future, when more interventions have been published, researchers may be able to categorize them into more homogenous groups in order to identify the most effective delivery of each specific type of intervention. Although the majority of studies reviewed were rated as having a low risk of bias, a large proportion of interventions came from studies with unclear or high risk of bias. Although the risk of bias had minimal effect on effect sizes, readers are cautioned to consider this, particularly when interpreting the pooled effect of GP endorsement which decreased when interventions from studies with high risk of bias were removed.

## Conclusion

Findings from the current review suggest that GP endorsement, telephone interaction, advance notification, and the issue of FIT over gFOBT kits are promising strategies for increasing uptake in mail-out screening programs. Further research is needed to understand the mechanisms underlying the success of these interventions and how they might be applied in different settings. Nevertheless, government and health organizations aiming to increase the general population uptake of FOBT screening for CRC will likely benefit from incorporating these strategies into future interventions. Lastly, it is important to note that the potential increases in uptake from current intervention strategies are modest. Novel or combined approaches to encourage CRC screening may be required if population-wide compliance is to be achieved.

## Supplementary information

**Additional file 1.** Contains the PRISMA checklist.

**Additional file 2.** Contains each search syntax entered for each database/source.

**Additional file 3.** Contains full table of extracted data from each study including author, date, sample size, age and country of participants, intervention type, comparison group, key findings and risk of bias.

**Additional file 4.** Contains a forest plot for each meta-analysis conducted on each intervention type (including those with interventions from high risk of bias studies included and excluded).

## Data Availability

Kit return data extracted from articles used in meta-analysis available on request from the corresponding author.

## Abbreviations

CI: Confidence interval
CRC: Colorectal cancer
FIT: Fecal immunochemical test
FOBT: Fecal occult blood test
gFOBT: Guaiac-based fecal occult blood test
GP: General practitioner
RR: Risk ratio

## References

[1] Parkin D (2002). World Health Organization cancer incidence in five continents Lyon. World Health OrganInt Agency Res Cancer.

[2] Government A, D.o.H.a. Aging (2005). The Austalian Bowel Cancer Screening Pilot Program and Beyond: Final Evaluation Report.

[3] Frazier A. Lindsay (2000). Cost-effectiveness of Screening for Colorectal Cancer in the General Population. JAMA.

[4] Kronborg O (2004). Randomized study of biennial screening with a faecal occult blood test: results after nine screening rounds. Scand J Gastroenterol.

[5] Scholefield J, et al. Nottingham trial of faecal occult blood testing for colorectal cancer: a 20-year follow-up. Gut. 2011; p. gutjnl-2011-300774. 10.1136/gutjnl-2011-300774 22052062

[6] Ananda S (2016). Survival impact of the Australian National Bowel Cancer Screening Programme. Intern Med J.

[7] Australian Institue of Health and Welfare. Analysis of bowel cancer outcomes for the National Bowel Cancer Screening Program. Australian Government, Canberra; 2014. Report No.: Cat. no. CAN 87.

[8] Navarro M (2017). Colorectal cancer population screening programs worldwide in 2016: an update. World J Gastroenterol.

[9] Swan H, Siddiqui AA, Myers RE. International colorectal cancer screening programs: population contact strategies, testing methods and screening rates. Pract Gastroenterol. 2012;36(8):20–9.

[10] Liberati A (2009). The PRISMA statement for reporting systematic reviews and meta-analyses of studies that evaluate health care interventions: explanation and elaboration. PLoS Med.

[11] Higgins JP (2011). The Cochrane Collaboration’s tool for assessing risk of bias in randomised trials. BMJ.

[12] Team RC (2017). R: A language and environment for statistical computing.

[13] Viechtbauer W (2010). Conducting meta-analyses in R with the metafor package. J Stat Softw.

[14] Viechtbauer W (2005). Bias and efficiency of meta-analytic variance estimators in the random-effects model. J Educ Behav Stat.

[15] Michael Borenstein, Hedges LV, Julian P. T. Higgins, Hannah R. Rothstein, Introduction to Meta-Analysis. 2009, United Kingdom: Wiley.

[16] Benton SC (2017). GP participation in increasing uptake in a national bowel cancer screening programme: the PEARL project. Br J Cancer.

[17] Cole SR (2002). Participation in screening for colorectal cancer based on a faecal occult blood test is improved by endorsement by the primary care practitioner. J Med Screen.

[18] Cole SR (2003). A randomised trial of the impact of new faecal haemoglobin test technologies on population participation in screening for colorectal cancer. J Med Screen.

[19] Cole SR (2007). An advance notification letter increases participation in colorectal cancer screening. J Med Screen.

[20] Coronado Gloria D., Rivelli Jennifer S., Fuoco Morgan J., Vollmer William M., Petrik Amanda F., Keast Erin, Barker Sara, Topalanchik Emily, Jimenez Ricardo (2017). Effect of Reminding Patients to Complete Fecal Immunochemical Testing: A Comparative Effectiveness Study of Automated and Live Approaches. Journal of General Internal Medicine.

[21] Denters MJ (2013). A feces collection paper does not enhance participation in a fecal immunochemical test-based colorectal cancer screening program: randomized clinical trial. Eur J Cancer Prev.

[22] Deutekom M (2010). Comparison of guaiac and immunological fecal occult blood tests in colorectal cancer screening: the patient perspective. Scand J Gastroenterol.

[23] Gupta S (2016). Financial incentives for promoting colorectal cancer screening: a randomized, comparative effectiveness trial. Am J Gastroenterol.

[24] Hewitson P, Ward AM, Heneghan C, Halloran SP, Mant D (2011). Primary care endorsement letter and a patient leaflet to improve participation in colorectal cancer screening: Results of a factorial randomised trial. Br J Cancer.

[25] Hirst Y, Skrobanski H, Kerrison RS, Kobayashi LC, Counsell N, Djedovic N (2017). Text-message Reminders in Colorectal Cancer Screening (TRICCS): A randomised controlled trial. Br J Cancer.

[26] Hughes K (2005). Guaiac versus immunochemical tests: faecal occult blood test screening for colorectal cancer in a rural community. Aust N Z J Public Health.

[27] King J (1992). Colorectal cancer screening: optimal compliance with postal faecal occult blood test. Aust N Z J Surg.

[28] King J, Fairbrother G, Thompson C, Morris DL (1994). Influence of socioeconomic status, ethnicity and an educational brochure on compliance with a postal faecal occult blood test. Aust N Z J Public Health.

[29] Libby G (2011). Pre-notification increases uptake of colorectal cancer screening in all demographic groups: a randomized controlled trial. J Med Screen.

[30] Lo SH (2014). Preformulated implementation intentions to promote colorectal cancer screening: a cluster-randomized trial. Health Psychol.

[31] McGregor LM, et al. Reducing the social gradient in uptake of the NHS colorectal cancer screening programme using a narrative-based information leaflet: a cluster-randomised trial. Gastroenterol Res Pract. 2016;2016. 10.1155/2016/3670150 PMC4812359 27069473

[32] Moss S, Mathews C, Day TJ, Smith S, Seaman HE, Snowball J (2017). Increased uptake and improved outcomes of bowel cancer screening with a faecal immunochemical test: Results from a pilot study within the national screening programme in England. Gut.

[33] Myers RE (1991). Behavioral interventions to increase adherence in colorectal cancer screening. Med Care.

[34] Neter Efrat, Stein Nili, Barnett-Griness Ofra, Rennert Gad, Hagoel Lea (2014). From the Bench to Public Health. American Journal of Preventive Medicine.

[35] O'Carroll RE (2015). Anticipated regret to increase uptake of colorectal cancer screening (ARTICS): a randomised controlled trial. Soc Sci Med.

[36] Robinson MHE (1994). Haemoccult screening for colorectal cancer: the effect of dietary restriction on compliance. Eur J Surg Oncol.

[37] Santare D (2015). Improving uptake of screening for colorectal cancer: a study on invitation strategies and different test kit use. Eur J Gastroenterol Hepatol.

[38] Van Roon AHC (2011). Advance notification letters increase adherence in colorectal cancer screening: a population-based randomized trial. Prev Med.

[39] Van Rossum LG (2008). Random comparison of guaiac and immunochemical fecal occult blood tests for colorectal cancer in a screening population. Gastroenterology.

[40] Verne J (1993). Self-administered faecal occult blood tests do not increase compliance with screening for colorectal cancer: results of a randomized controlled trial. Eur J Cancer Prev.

[41] Wardle J, von Wagner C, Kralj-Hans I, Halloran SP, Smith SG, McGregor LM (2016). Effects of evidence-based strategies to reduce the socioeconomic gradient of uptake in the English NHS Bowel Cancer Screening Programme (ASCEND): four cluster-randomised controlled trials. The Lancet.

[42] Watson J (2013). Use of research questionnaires in the NHS bowel Cancer screening Programme in England: impact on screening uptake. J Med Screen.

[43] White B (2015). Piloting the impact of three interventions on guaiac faecal occult blood test uptake within the NHS bowel cancer screening programme. Biomed Res Int.

[44] Zajac IT (2010). Endorsement by the primary care practitioner consistently improves participation in screening for colorectal cancer: a longitudinal analysis. J Med Screen.

[45] Zubero MB, et al. Population-based colorectal cancer screening: comparison of two fecal occult blood test. Front Pharmacol. 2014;4:175. 10.3389/fphar.2013.00175 PMC3887272 24454288

[46] Hewitson P (2011). Primary care endorsement letter and a patient leaflet to improve participation in colorectal cancer screening: results of a factorial randomised trial. Br J Cancer.

[47] Wardle J (2016). Effects of evidence-based strategies to reduce the socioeconomic gradient of uptake in the English NHS bowel cancer screening programme (ASCEND): four cluster-randomised controlled trials. Lancet.

[48] Neter E (2014). From the bench to public health: population-level implementation intentions in colorectal cancer screening. Am J Prev Med.

[49] Arnold CL (2016). Final results of a 3-year literacy-informed intervention to promote annual fecal occult blood test screening. J Community Health.

[50] Moss Sue, Mathews Christopher, Day T J, Smith Steve, Seaman Helen E, Snowball Julia, Halloran Stephen P (2016). Increased uptake and improved outcomes of bowel cancer screening with a faecal immunochemical test: results from a pilot study within the national screening programme in England. Gut.

[51] Coronado GD (2011). Effectiveness of a clinic-based colorectal cancer screening promotion program for underserved Hispanics. Cancer.

[52] Coronado GD (2018). Effect of reminding patients to complete fecal immunochemical testing: a comparative effectiveness study of automated and live approaches. J Gen Intern Med.

[53] Hirst Y, et al. Text Reminders in Colorectal Cancer Screening (TRICCS): Protocol for a randomised controlled trial. BMC Public Health. 2016;16(1):74. 10.1186/s12889-016-2733-6 PMC4727285 26809344

[54] Pennbridge J, Moya R, Rodrigues L (1999). Questionnaire survey of California consumers' use and rating of sources of health care information including the internet. West J Med.

[55] Närhi U (2007). Sources of medicine information and their reliability evaluated by medicine users. Pharm World Sci.

[56] Khoo K (2008). Health information seeking by parents in the internet age. J Paediatr Child Health.

[57] Duffy Stephen W, Myles Jonathan P, Maroni Roberta, Mohammad Abeera (2016). Rapid review of evaluation of interventions to improve participation in cancer screening services. Journal of Medical Screening.

[58] Reynolds LM (2013). Disgust and behavioral avoidance in colorectal cancer screening and treatment: a systematic review and research agenda. Cancer Nurs.

[59] Schreuders EH (2015). Colorectal cancer screening: a global overview of existing programmes. Gut.

[60] Rücker G, Schwarzer G, Carpenter JR, Schumacher M (2008). Undue reliance on I 2 in assessing heterogeneity may mislead. BMC Med Res Methodol.

