# Supplementary material for: Strategies for increasing participation in mail-out colorectal cancer screening programs: a systematic review and meta-analysis
Source: Syst Rev. 2019 Nov 4;8:257. doi: 10.1186/s13643-019-1170-x (PMC6827213; doi:10.1186/s13643-019-1170-x)
Supplement: Supplementary file 2 — Additional file 2. Contains each search syntax entered for each database/source. [file 13643_2019_1170_MOESM2_ESM.docx]

Additional File 2. Syntax and results for each database search conducted on the 6/03/2017, 17/05/2017, and 10/03/2018

**Pubmed (n= 1134)**

((("fecal occult blood"[Title/Abstract] OR "faecal occult blood"[Title/Abstract] OR FOBT[Title/Abstract] OR "fecal immunochemical test" OR "faecal immunochemical test" [Title/Abstract])) AND (participat*[Title/Abstract] OR adher*[Title/Abstract] OR uptake[Title/Abstract] OR return [Title/Abstract] OR complian* [Title/Abstract]))

**Scopus, accessed through Elsevier (n= 1387)**

(AND TITLE-ABS-KEY "fecal occult blood" OR "faecal occult blood" OR fobt OR "fecal immunochemical test" OR "faecal immunochemical test" ) AND TITLE-ABS-KEY ( participat* OR adher* OR uptake OR return OR complian* ) )

**PsycInfo, accessed through EBSCOhost (n= 162)**

TI ( "fecal occult blood" OR "faecal occult blood" OR fobt OR "fecal immunochemical test" OR "faecal immunochemical test" ) AND TI ( participat* OR adher* OR uptake OR return OR complian* ) OR AB ( "fecal occult blood" OR "faecal occult blood" OR fobt OR "fecal immunochemical test" OR "faecal immunochemical test" ) AND AB ( participat* OR adher* OR uptake OR return OR complian* ) OR KW ( "fecal occult blood" OR "faecal occult blood" OR fobt OR "fecal immunochemical test" OR "faecal immunochemical test" ) AND KW ( participat* OR adher* OR uptake OR return OR complian* )**CINAHL, accessed through EBSCOhost (n= 483)**

(TI ( "fecal occult blood" OR "faecal occult blood" OR FOBT OR "fecal immunochemical test" OR "faecal immunochemical test" ) OR AB ( "fecal occult blood" OR "faecal occult blood" OR FOBT OR "fecal immunochemical test" OR "faecal immunochemical test" )) AND (TI ( participat* OR adher* OR uptake OR return OR complian* ) OR AB ( participat* OR adher* OR uptake OR return OR complian* )) **Google Scholar (n= 44)**

allintitle: "fecal occult blood" OR "feacal occult blood" OR FOBT OR "fecal immunochemical test" OR "faecal immunochemical test" AND participate OR participation OR uptake OR return OR adhere OR adherence OR compliance OR compliant

**Proquest Theses and Dissertations (n= 3)**

ti("faecal occult blood" OR "fecal occult blood" OR fobs OR "fecal immunochemical test" OR "faecal immunochemical test") AND ti(participat* OR uptake OR return OR complian* OR adher*) OR ab("faecal occult blood" OR "fecal occult blood" OR fobs OR "fecal immunochemical test" OR "faecal immunochemical test") AND ab(participat* OR uptake OR return OR complian* OR adher*)

**Hand searches (n=10)- conducted after identifying full text articles for review**

Articles were located through reference lists of included studies or ‘cited by’ function on Google Scholar (n=6). In addition, a combination of key terms were entered into several Google searches (e.g., “intervention to increase return of FOBt kits”) in order to search for any non-academic (e.g., government reports) that may address the question (n=0).
