# Supplementary material for: Strategies for increasing participation in mail-out colorectal cancer screening programs: a systematic review and meta-analysis
Source: Syst Rev. 2019 Nov 4;8:257. doi: 10.1186/s13643-019-1170-x (PMC6827213; doi:10.1186/s13643-019-1170-x)
Supplement: Supplementary file 3 — Additional file 3. Contains full table of extracted data from each study including author, date, sample size, age and country of participants, intervention type, comparison group, key findings and risk of bias. [file 13643_2019_1170_MOESM3_ESM.docx]

Additional file 3. Data extraction table by intervention type

| **First author and year** | **Description of sample (n)** | **Intervention** | **Design**  **(Control / Reference)** | **Findings^¥^** | **Risk of bias** |
| --- | --- | --- | --- | --- | --- |
| **GP Endorsement** | | | | | |
| Benton et al., 2017 | 60 – 74 yr. olds from GP records, UK (n= 24,736) | Reminder letter sent after invitation pack from GP endorsing screening***** | Non-RCT  (Standard reminder letter from screening organization) | The issue of a reminder letter to non-responders significantly increased participation compared to a standard reminder letter RR = 1.09 (CI = 1.06 – 1.11). | High |
| Cole et al., 2002 (1) | 50 + yr. olds from GP records and electoral roll, Australia (n=1800) | Letter from GP with invitation pack endorsing screening***** | RCT  (Standard invitation letter sent from central screening service) | A letter from a GP of recent contact with a screening offer in the form of a personalized letter of invitation achieves significantly better participation than does the same letter from a centralized screening unit RR = 1.18 (CI = 1.04 – 1.35). | Low |
| Hewitson et al., 2011 | 60 – 75 yr. olds from  GP records, UK (n=1288) | Letter from GP with invitation pack endorsing screening***** | RCT  (Standard invitation letter from screening organization) | Including an endorsement letter from each patient’s GP could increase participation in the English Bowel Cancer Screening Program RR = 1.11 (CI = 1.01 – 1.22). | Low |
| King et al., 1992 | 45-75 yr. olds from GP records, Australia (n=793) | Letter from GP with invitation pack endorsing screening***** | Non-RCT  (Generic invitation letter from researchers) | A general practitioner letter attached to the FOBT kit results in higher screening compliance, compared to a generic invitation letter RR = 2.19 (CI = 1.67 – 2.87). | High |
| Wardle et al., 2015 | 60 – 74 yr. olds from population database of GP patients, UK (n=265434) | Letter from GP with invitation pack endorsing screening***** | Cluster RCT  (Standard invitation from screening organization) | General practice endorsement significantly increased overall uptake, but the effect size was small RR = 1.01 (CI = 1.01 - 1.02). | Low |
| Cole et al., 2002 (2) | 50 + yr. olds from GP records and electoral roll, Australia (n=1800) | Letter from GP *practice* with invitation pack endorsing screening***** | RCT  (Standard invitation letter sent from central screening service) | Associating a GP *practice* of recent contact with an invitation to screen achieves better participation than an invitation from a centralized screening unit RR 1.27 (CI = 1.12 – 1.44). | Low |
| Zajac et al., 2010 (1) | Sample from Cole et al., 2002 study less attrition over 3 subsequent rounds  (n= R2: 1642, R3: 1290, R4: 1361) | Letter from GP  *practice*  with invitation pack endorsing screening | RCT  (Standard invitation letter sent from central screening service) | Associating a GP *practice* of recent contact with an invitation to screen did not achieve better participation than an invitation from a centralized screening unit over time R2: RR = 1.12 (CI = 0.98 – 1.27), R3: RR = 1.11 (CI = 0.97 – 1.27), R4: RR = 1.19 (1.04 – 1.37) | High |
| Zajac et al., 2010 (2) | Sample from Cole et al., 2002 study less attrition over 3 subsequent rounds  (n= R2:1637, R3: 1287, R4: 1364) | Letter from GP with invitation pack endorsing screening***** | RCT  (Standard invitation letter sent from central screening service) | A letter from a GP of recent contact with a screening offer in the form of a personalized letter of invitation achieves significantly better participation than does the same letter from a centralized screening unit and this remains somewhat stable over time R2: RR = 1.25 (CI = 1.11 – 1.41), R3: RR = 1.18 (CI = 1.04 – 1.35), R4: RR = 1.33 (1.17 – 1.51). | High |
| **Behavior priming** | | | | |  |
| Cole et al., 2007 (1) | 50-74 yr. olds from electoral roll, Australia (n=1200) | Photographs and advocacy statements from lay persons within standard invitation letter. | RCT  ( Standard invitation letter containing CRC prevention and value and ease of screening) | Lay advocacy strategies did not improve screening participation RR = 0.91 (CI = 0.79 – 1.05). | Unclear |
| Lo et al., 2014 | 60-69 yr. olds from national screening program, UK (n=23180) | Pre-formulated implementation intentions inserted into standard leaflet | RCT  (Standard leaflet) | Pre-formulated implementation intentions did not increase CRC screening uptake RR = 0.98 (CI = 0.95 – 1.01). | Unclear |
| Neter et al., 2013 | 50-74 yr. olds from medical insurance records, Israel (n=27633) | Placing ‘if then’ statements in leaflet to invoke implementation intentions***** | RCT  (Standard invitation) | Implementation intention technique is useful in increasing adherence to CRC screening RR = 1.05 (CI = 1.04 – 1.07). | Low |
| Cole et al., 2007 (2) | 50-74 yr. olds from electoral roll, Australia (n=1200) | Positively framed information about CRC risks within standard invitation letter | RCT  (Standard invitation letter containing CRC prevention and value and ease of screening) | Given participants extra information on risk does not significantly improve uptake RR = 1.02 (CI = 0.89 – 1.17). | Unclear |
| Myers et al., 1991 | 50 -74 yr. olds from health maintenance organization, US (n=2201) | Loss framed messages in all communications with participants | RCT  (Gain framed messages in all communications with participants) | More subjects receiving the "loss" messages returned tests, but the size of this difference was small and not statistically significant RR = 0.91 (0.82 – 1.02). | Unclear |
| O'Carroll et al., 2015 | 50 – 74 yr. olds from national screening program, Scotland (n=39,762) | Invitation pack includes survey with questions to provoke anticipated regret | RCT  ( Invitation includes survey without questions to provoke anticipated regret) | The anticipated regret intervention had no simple effect on FOBT uptake RR = 1.01 (CI = 0.99 – 1.03). | Low |
| **Print materials added to kit** | | | | |  |
| Hewitson et al., 2011 | 60 – 75 yr. olds from  GP practices, UK (n=1288) | Enhanced procedural instruction leaflet with tips and advice on how to collect, store and return samples***** | RCT  (All groups that did not receive enhanced leaflet, i.e., standard invite and GP letter) | Including an explicit procedural leaflet could increase participation in the English Bowel Cancer Screening Program RR = 1.12 (CI = 1.01 – 1.23). | Low |
| Wardle et al., 2015 (1) | 60 – 74 yr. olds from population database of GP patients, UK (n=168480) | Enhanced reminder letter including “a reminder to you” banner***** | Cluster RCT  (Standard reminder letter) | The enhanced reminder letter slightly increased overall uptake RR = 1.03 (CI = 1.01 – 1.05). | Low |
| King et al., 1994 | 45-75 yr. olds from GP records, Australia (n=1281) | Low literacy booklet with graphics and risk information sent with invitation pack | Cluster RCT  (Standard invitation pack) | A brochure, designed to increase compliance with CRC testing, had no significant effect in Australian samples RR = 1.01 (CI = 0.92 – 1.12). | Unclear |
| Libby et al., 2011 | 50 – 74 yr. olds from national screening program, Scotland (n=39,966) | “Know the facts” information booklet included with advance notification letter | RCT  (Advance notification without booklet and followed by standard invitation pack) | Participants that received information booklets were not more likely to participate than those who did not RR = 0.99 (CI = 0.98 – 1.01). | Low |
| Myers et al., 1991 | 50 -74 yr. olds from health maintenance organization, US (n=900) | ColoRecord booklet with information on the efficacy of FOB testing included with invitation pack (+ reminder call) | RCT  (Standard invitation pack without booklet + reminder call) | The inclusion of a ColoRecord booklet in the screening kit did not lead to increased test returns RR = 1.01 (CI = 0.85 to 1.20). | Unclear |
| White et al., 2015 | 60 -74 yr. olds from national screening program, UK (n=197840) | CRUK booklet emphasizing efficacy, privacy, ease of use and acceptability of text sent with invitation pack | Non-RCT  (Standard invitation pack without booklet) | Participants that received information booklets with their screening kits were not more likely to participate than those who did not RR = 0.98 (CI = 0.96 – 1.01). | Unclear |
| McGregor et al., 2016 | 60 – 74 yr. olds from national screening program, UK (n=150,417) | A narrative leaflet with quotes, photographs and stories of previous participants sent with the invitation pack | Cluster-RCT  (Standard invitation pack with leaflet without narratives) | The inclusion of a narrative leaflet on outcomes significantly *decreased* FOBT uptake RR = 0.97 (CI = 0.96 - 0.98). | Low |
| Wardle et al., 2015 (2) | 60 – 74 yr. olds from population database of GP patients, UK (n=150417) | A narrative leaflet with presenting stories of previous participants sent with the invitation pack | Standard invitation pack without narrative leaflet  Study design: Cluster RCT | The inclusion of a narrative leaflet on outcomes significantly *decreased* FOBT uptake RR = 0.97 (CI = 0.96 – 0.98). | Low |
| Wardle et al., 2015 (3) | 60 – 74 yr. olds from population database of GP patients, UK (n=163525) | A “gist” leaflet: Simplified version of the screening information leaflet designed for low literacy and numeracy readers | Cluster RCT  (Standard invitation pack with standard information leaflet) | The gist and leaflet interventions had no effect on uptake RR 1.01 (CI = 1.00 – 1.02). | Low |
| O'Carroll et al., 2015 | 50 – 74 yr. olds from national screening program, Scotland (n=59,366) | Invitation pack included a survey including health locus of control questions | RCT  (Standard invitation pack without survey) | The inclusions of a survey in a screening kit did not significantly affect screening uptake RR = 1.00 (CI = 0.98 – 1.01). | Low |
| Watson et al., 2013 | 60 -74 yr. olds from national screening program, UK (n=11579) | Survey with medical and lifestyle questions send within a few days of invitation pack | RCT  (Standard information pack without survey) | The inclusion of a survey with the kit significantly *decreased* FOBT uptake RR = 0.91 (CI = 0.88 – 0.94). | Low |
| **Simplified test procedure** | | | | |  |
| Cole et al., 2003 (1) | 50-69 yr. olds from electoral roll, Australia (n=1818) | Kit that requires participant to collect 2 samples with brush***** | RCT  (Kits that requires participant to collect 3 samples using spatula) | Reducing the required stool samples from two to three, combined with a brush application, significantly increases participation RR = 1.47 (CI = 1.28 – 1.68). | Low |
| Denters et al., 2013 | 50 -75 yr. olds from national screening program, Netherlands (n=10265) | Kit that includes feces collection paper for the toilet bowl | RCT  (Kit that does not include feces collection paper) | A feces collection paper does not increase participation rates in FIT-based CRC cancer screening RR = 0.99 (CI = 0.96 – 1.03). | Unclear |
| Cole et al., 2003 (2) | 50-69 yr. olds from electoral roll, Australia (n=1818) | Instructions do not include dietary restrictions ***** | RCT  (Instructions do include dietary restrictions) | Participants not required to conform to dietary restrictions are more likely to participate in mail-out bowel cancer screening, rather than a those that are RR = 1.50 (CI = 1.27 – 1.76). | Low |
| Deutekom et al., 2010 | 50 -75yr. olds from municipal records, Netherlands (n= 20,623) | Test involves one sample collected with brush (FIT)***** | RCT  (Test involves three samples collected with cardboard stick; gFOBT) | The participation rate was significantly higher in those who were required to collect one sample with a brush (FIT) than those who were required to collect three samples with a cardboard stick (gFOBT) RR = 1.27 (CI = 1.24 – 1.30). | Low |
| Hughes et al., 2005 | 50-74 yr. olds from GP records, rural Australia (n=3358) | Test involves two samples collected with brush and no diet restrictions (FIT)***** | Cluster RCT  (Test involves three samples collected with spatula and includes diet restrictions; gFOBT) | The participation rate was significantly higher for those who were required to collect two sample with a brush and no dietary restrictions (FIT) than those who were required to collect three samples with a spatula and conform to dietary restrictions (gFOBT) RR = 1.28 (CI = 1.15 – 1.43) | High |
| Moss et al., 2017 | 60 – 74 yr. olds from national screening program, UK (n=116,7017) | Participants issued FIT***** | Cluster RCT  (Participants issued gFOBT) | The use of FIT (rather than gFOBT) resulted in significantly higher uptake RR = 1.12 (CI = 1.11 – 1.13). | Low |
| Santare et al., 2015 (1) | 50 – 74 yr. olds from population, screening and cancer registries, Latvia (n=15,000) | Instructions do not include dietary restrictions (FIT)***** | RCT  (Instructions do include dietary restrictions; gFOBT) | Participants not required to conform to dietary restrictions (FIT) are more likely to participate in mail-out bowel cancer screening, rather than a those that are (gFOBT) RR = 1.48 (CI = 1.41 – 1.55). | Unclear |
| van Rossum et al., 2008 | 50 – 75 yr. olds from municipal database, Netherlands (n=20,623) | Sample from one bowel motion required, collected in tube (FIT)***** | RCT  (Sample from three bowel motions required collected on cards; gFOBT) | Analysis revealed a significantly higher participation rate for the FIT requiring collection in tube from one bowel motion compared to gFOBT requiring collection on card from three bowel motions RR = 1.28 (CI = 1.24 – 1.31) | Low |
| Santare et al., 2015 (2) | 50 – 74 yr. olds from population, screening & cancer registries, Latvia (n=9770) | Kit includes round test tube with two caps (OC Sensor)***** | RCT  (Kit includes flat test tube with one cap; FOB Gold) | The use of OC-Sensor compared to FOB Gold, was significantly associated with higher uptake RR = 1.06 (CI = 1.02 – 1.11). | Unclear |
| Zubero et al., 2014 | 50 – 69 yr. olds from Basque screening program, Spain (n=37,999) | Kit includes round test tube with two caps (OC Sensor)***** | Non-RCT  (Kit includes flat test tube with one cap (FOB Gold) | The use of OC-Sensor resulted in a consistently increased absolute participation rate compared to FOB Gold, but the effect was not statistically significant RR = 1.05 (CI = 1.03 – 1.06). | Low |
| White et al., 2015 | 60 -74 yr. olds from national screening program, UK (n=19382) | Kit included gloves and feces collection paper for toilet bowl***** | Non-RCT  (Standard kit) | Participants who received gloves and collection paper with their screening kit were significantly more likely to participate in the program RR = 1.05 (CI = 1.02 – 1.09). | Unclear |
| King et al., 1992 | 45-75 yr. olds from GP records, Australia (n=593) | Instructions do not include dietary restrictions | Non-RCT  (Instructions do include dietary restrictions) | Screening uptake was not significantly lower when participants were instructed to restrict their diets RR = 1.05 (CI = 0.88 – 1.25). | High |
| Robinson et al. 1994 (1) | 50-74 yr. old from one GP practice’s records, UK (n=153) | Instructions do not include dietary restrictions ***** | RCT  (Instructions do include dietary restrictions) | In a British population compliance, gFOBT screening uptake is adversely affected by the dietary restrictions RR = 1.42 (CI = 1.09 - 1.83). | Unclear |
| Robinson et al. 1994 (2) | 50-74 yr. old from one GP practice’s records, UK (n=153) | Testing completed over 6 days | RCT  (Testing completed over 3 days) | Increasing the length of time to complete test does not significantly increase uptake RR = 1.02 (CI = 0.80 – 1.31). | Unclear |
| Verne et al., 1993 (1) | 40 – 75 yr. olds from large GP practice records, UK (n=1842) | Instructions do not include dietary restrictions | RCT  (Instructions do include dietary restrictions) | Dietary restrictions did not reduce compliance significantly RR = 1.05 (CI = 0.96 – 1.15) | Unclear |
| Verne et al., 1993 (2) | 40 – 75 yr. olds from large GP practice records, UK (n=1233) | Results of test are self-analyzed at home | RCT  (Results posted to laboratory for analysis) | Participants receiving self-analyzed kits were not significantly more like to comply with screening than those receiving a laboratory analyzed kit RR = 1.03 (CI = 0.92 – 1.15). | Unclear |
| Verne et al., 1993 (3) | 40 – 75 yr. olds from large GP practice records, UK (n=1243) | Stool sample collected using a wipe | RCT  (Standard stool sample collection from bowl) | Participants who received kits whereby stool sample was collected using a wipe were not more likely to participate in screening than those using a bowl collection method RR = 1.06 (CI = 0.95 – 1.18). | Unclear |
| **Telephone contact** | | | | |  |
| Myers et al., 1991 (1) | 50 -74 yr. olds from health maintenance organization, US (n=1150) | An instruction call to assist recipients with kit completion | RCT  (All groups that did not receive an instruction call***)** | Adherence rates were significantly increased in groups that received telephone instructions RR = 1.29 (CI = 1.12 – 1.49). | Unclear |
| Myers et al., 1991 (2) | 50 -74 yr. olds from health maintenance organization, US (n=2201) | Participants who did not return kit within first 30 days received a live telephone reminder call | RCT  (All groups that did not receive a reminder call***)** | Adherence rates were significantly increased in groups that received a reminder telephone call RR = 1.34 (CI = 1.13 – 1.60). | Unclear |
| Coronado et al., 2018 (2) | 50-75 yr olds from selected GP practices, US (n=542) | Participants who did not return kit within first 3 weeks received a live telephone reminder call***** | RCT  (All groups that did not receive a live reminder call*) | A live reminder call is significantly effective in increasing uptake RR = 1.31 (CI = 1.12 – 1.53). | Low |
| Coronado et al., 2018 (3) | 50-75 yr olds from selected GP practices, US (n=571) | Participants who did not return kit within first 3 weeks received an automatic telephone reminder call | RCT  (All groups that did not receive an automatic reminder call) | Automated phone calls were not successful in increasing participation compared to a reminder letter RR = 1.01 (0.86 – 1.19). | Low |
| **Digital reminders** | | | | |  |
| Coronado et al., 2018 (1) | 50-75 yr olds from selected GP practices, US (n=475) | Participants who did not return kit within first 3 weeks received a reminder email | RCT  (Participants who did not return kit within first 3 weeks received a standard reminder letter) | Email reminders did not increase participation RR = 0.87 (CI = 0.62 – 1.23). | High |
| Coronado et al., 2018 (4) | 50-75 yr olds from selected GP practices, US (n=2010) | Participants who did not return kit within first 3 weeks received a reminder text message | RCT  (All groups that did not receive a reminder text message) | Text messages reminders as opposed to a reminder letter did not improve uptake RR = 0.86 (CI = 0.72 – 1.01) | Low |
| Hirst et al., 2017 | 60 – 74 yr olds from GP records, UK (n=8266) | Text message from GP endorsing bowel screening sent with invitation kit | RCT  (Usual invitation process without a text message) | Endorsement text from the recipient’s GP did not improve uptake overall RR = 1.01 (CI = 0.96 – 1.07). However, in a sub-group of new invitees, the text had a significant positive effect 1.16 (1.02 – 1.32). | Low |
| **Advance notification** | | | | |  |
| Cole et al., 2007 (3) | 50-74 yr. olds from electoral roll, Australia (n=1200) | Advanced notification letter two weeks prior to receiving standard invitation pack***** | RCT  (Standard invitation pack only) | Advance notification significantly increased screening participation RR = 1.22 (CI =1.08 – 1.39). | Unclear |
| Libby et al., 2011 (2) | 50 – 74 yr. olds from national screening program, Scotland (n=39,963) | Advanced notification letter sent prior to standard invitation pack***** | RCT  (Standard invitation pack only) | Pre-notification is an effective method of increasing uptake in colorectal cancer screening RR = 1.09 (CI = 1.08 – 1.11). | Low |
| Santare et al., 2015 (3) | 50 – 74 yr. olds from population, screening and cancer registries, Latvia (n=15,000) | Advanced notification letter two weeks prior to receiving standard invitation pack***** | RCT  (Standard invitation pack only) | Advanced notification letters were significantly associated with higher participation RR = 1.08 (CI = 1.04 – 1.12). | Unclear |
| van Roon, 2011 | 50 – 74 yr. olds from municipal database, Netherlands (n=5000) | Advanced notification letter two weeks prior to receiving standard invitation pack***** | RCT  (Standard invitation pack only) | Sending an advance notification letter significantly increases adherence RR 1.06 (CI = 1.01 – 1.11). | Unclear |
| **Other interventions** | | | | | |
| Gupta et al., 2016 | 50 – 64 yr. olds from health network register, US (n=8565) | Invitation recipients offered a $5 or $10 retail voucher for returning the kit. | RCT  (Standard invitation without incentive) | Financial incentives, in the amount of $5 or $10 offered in exchange for responding to mailed invitation to complete FIT, do not impact CRC screening completion RR =1.02 (CI = 0.95 – 1.09). | Low |
| White et al., 2015 | 60 -74 yr. olds from national screening program, UK (n=29800) | Advertising for screening program appeared on pharmacy bags, bus stops, GP practices and local press in recipients’ neighborhoods* | Non-RCT  (Recipients living in areas not exposed to advertisement) | Participants exposed to advertisements for CRC screening were more likely to participate in the national screening program RR = 1.08 (CI = 1.05 – 1.14). | High |

All “invitations packs” included an FOBT kit. Intervention groups were compared with either a control group or appropriate reference group in order to obtain the most precise estimate of the unique effect of each intervention. ^ Where risk ratios were not reported, they have been calculated based on participation rates provided in article; ¥ Original author interpretations are presented where possible, except when further analyses were conducted using study data and interpreted by the current authors; * intervention had significant positive effect on uptake p<.05
