# Supplementary material for: Strategies for increasing participation in mail-out colorectal cancer screening programs: a systematic review and meta-analysis
Source: Syst Rev. 2019 Nov 4;8:257. doi: 10.1186/s13643-019-1170-x (PMC6827213; doi:10.1186/s13643-019-1170-x)
Supplement: Supplementary file 4 — Additional file 4. Contains a forest plot for each meta-analysis conducted on each intervention type (including those with interventions from high risk of bias studies included and excluded). [file 13643_2019_1170_MOESM4_ESM.docx]

Additional file 4. Plots of pooled effect sizes for each intervention type.

**Interventions from all studies included:**


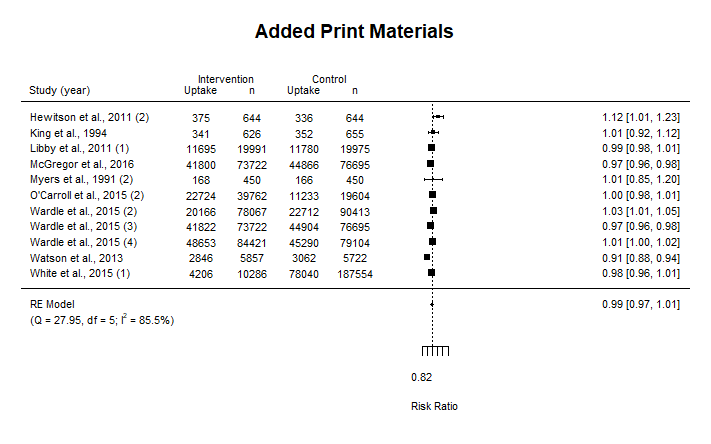

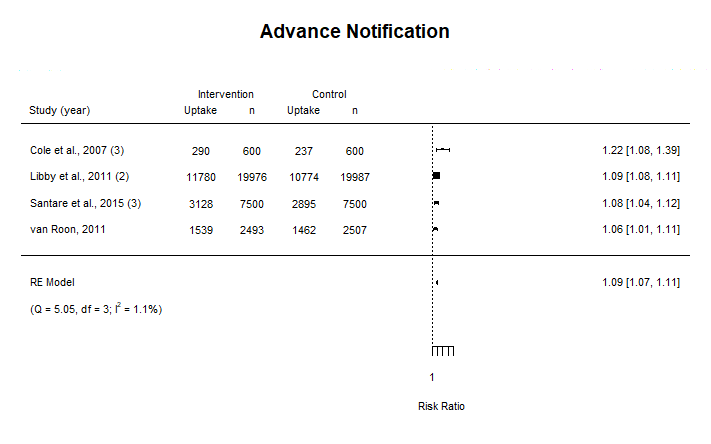

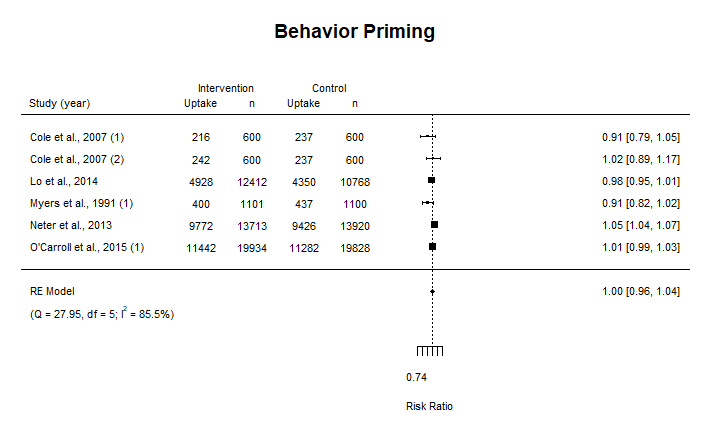

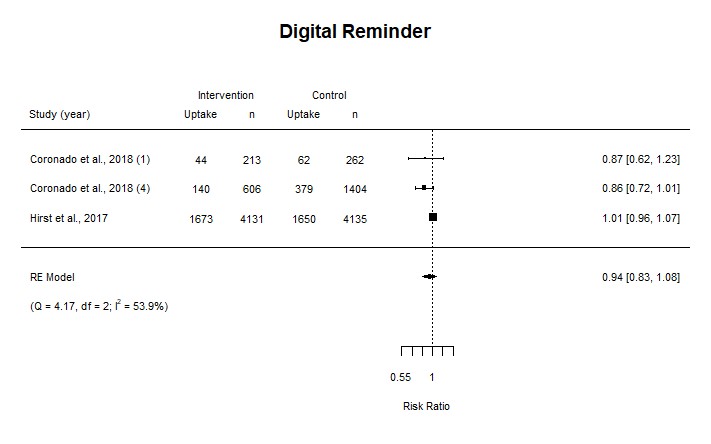

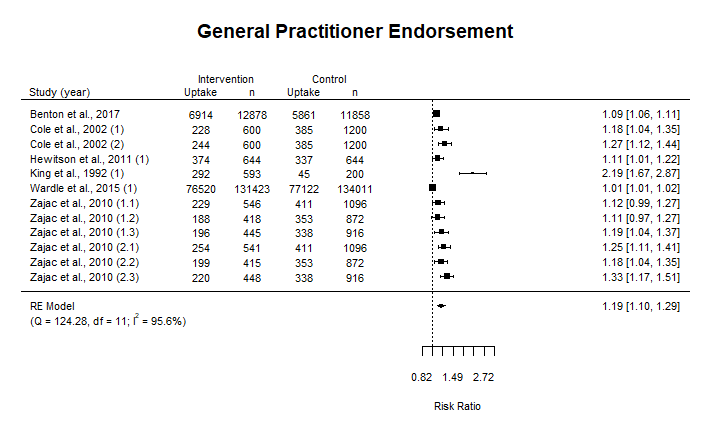

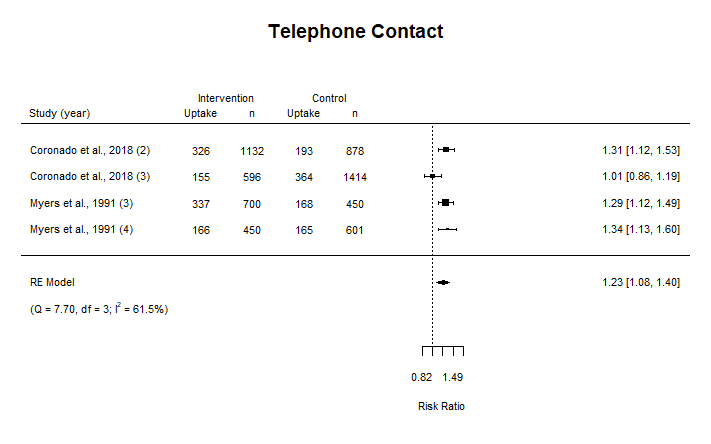


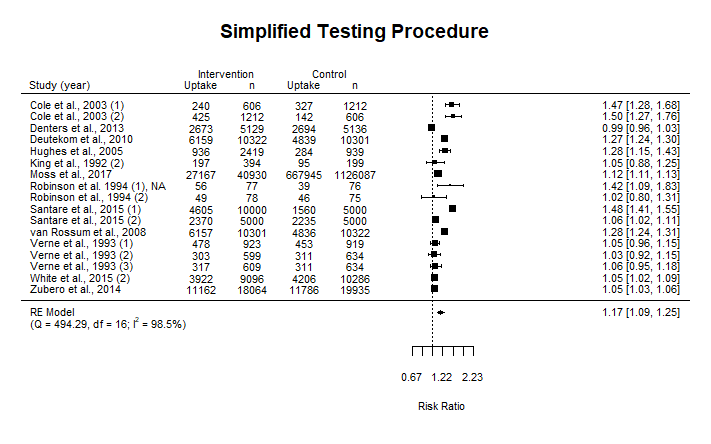


**Interventions from high risk studies removed:**

**
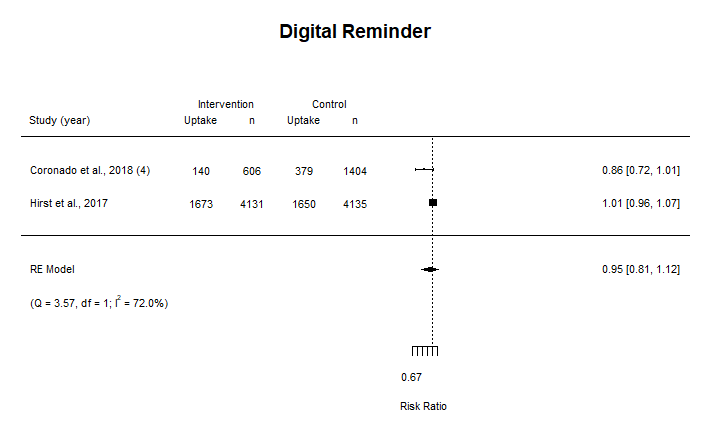

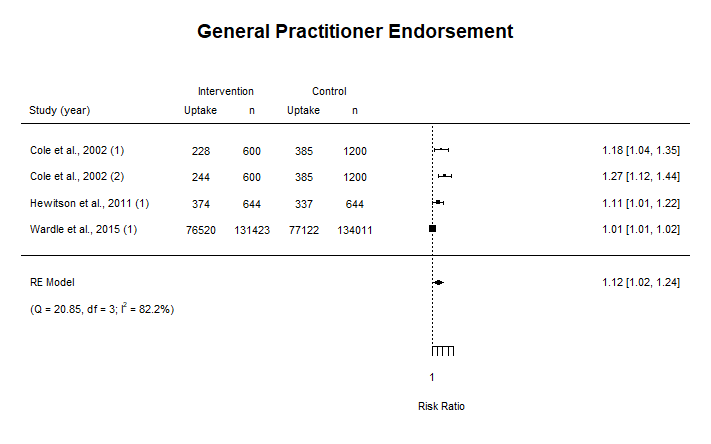
**

**
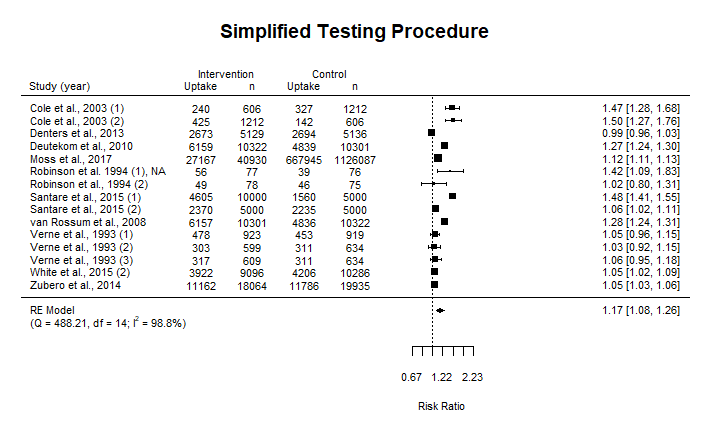
**
